# How to Assess the Degree of Pulmonary Congestion in Patients with Congestive Heart Failure

**DOI:** 10.3390/jcm12082889

**Published:** 2023-04-15

**Authors:** Teruhiko Imamura

**Affiliations:** Second Department of Internal Medicine, University of Toyama, 2630 Sugitani, Toyama 930-0194, Japan; teimamu@med.u-toyama.ac.jp; Tel.: +81-76-434-2281; Fax: +81-76-434-5026

With the introduction of several novel medications, including angiotensin receptor neprilysin inhibitors and sodium-glucose cotransporter 2 inhibitors, in addition to conventional beta-blockers and mineralocorticoid receptor antagonists, mortality and morbidity in patients with heart failure improved significantly [1]. Modulators of other pathways, including ivabradine and vericiguat, have recently been introduced and are being used in daily practice to further improve patient outcomes [1]. Nevertheless, the management of pulmonary congestion remains an unmet need that affects patients’ prognosis and quality of life [2].

The presence of pulmonary congestion impairs patients’ quality of life, particularly in those with frailty, sarcopenia, and malnutrition [1]. Residual pulmonary congestion at index discharge is associated with worse clinical outcomes, even if the amount is trivial. In addition, a recent study showed that even sub-clinical rather than symptomatic congestion was associated with right ventricular dysfunction and worse clinical outcomes [3].

One of the challenges in the management of pulmonary congestion is the lack of a gold standard to accurately quantify the degree of fluid volume [4]. Physical examination is one of the classic methods to assess body fluid distribution. However, the physical examination has limitations in estimating invasively measured pulmonary artery wedge pressure. Invasive right heart catheterization is used to assess body fluid distribution, including pulmonary artery wedge pressure, but may not be indicated in all heart failure patients because of its invasiveness [5]. Other modalities are clinically used to assess pulmonary congestion, including chest X-rays and the measurement of plasma B-type natriuretic peptide levels. However, the results of these modalities often require an expert interpretation.

Remote dielectric sensing (ReDS) is a recently introduced technology that uses electromagnetic energy to non-invasively and rapidly quantify the amount of lung fluid without the need for expert technique [4]. Its clinical utility has been validated by offering a comparison with other conventional modalities. ReDS values, which are displayed on the screen of the ReDS system monitor and represent the percentage of the lung fluid amount, had a moderate correlation with the percentage of lung fluid distribution calculated by computed tomography [4]. Chest-computed tomography is performed during inspiratory breathing, while the ReDS values are measured during natural breathing. The percentage of lung fluid amount may vary depending on the respiratory state. There was a moderate correlation between ReDS values and pulmonary artery wedge pressure [5]. The discrepancy between the two modalities is reasonable because each modality assesses pulmonary congestion in a different way. The right heart catheterization assesses intra-vascular congestion and ReDS technology assesses both intra-vascular congestion and tissue congestion. Some patients have increased intra-cardiac pressure without lung fluid amount. For example, pulmonary artery wedge pressure is elevated in the absence of pulmonary congestion in patients with a low cardiac output. Chest X-ray and lung ultrasound are clinically practical tools for distinguishing severe pulmonary congestion [6]. ReDS values and modalities showed moderate correlations, especially in patients with severe pulmonary congestion [7]. ReDS technology could stratify the degree of lung fluid amount in patients with mild pulmonary congestion by displaying the quantified ReDS values, whereas these modalities could not stratify those with mild pulmonary congestion. 

I am not denying the usefulness of these conventional methods. Each modality that includes a ReDS system has advantages and limitations. We can choose appropriate modalities, sometimes in combination, according to each clinical situation. Chest echocardiography is a good practical tool for assessing severe pulmonary congestion. However, it cannot accurately quantify the degree of mild congestion. Moreover, chest X-ray is an essential tool for assessing pulmonary congestion. It can visualize the thoracic anatomy and help us to assess other thoracic diseases. On the contrary, it cannot accurately quantify the degree of mild congestion. They may not be appropriate to adjust the dose of diuretics in patients with mild congestion. Plasma B-type natriuretic peptide is, needless to say, an essential method for estimating intra-cardiac pressure. However, there are several confounding factors, including obesity and renal dysfunction. Furthermore, an elevated B-type natriuretic peptide does not necessarily indicate the existence of pulmonary congestion, e.g., in patients with low cardiac output syndrome. The ReDS system can quantify the lung fluid. However, as detailed below, it cannot distinguish pulmonary congestion from other lung diseases that accompany lung fluid retention. This technology is more useful for screening out pulmonary congestion or tracking the degree of pulmonary congestion. 

The clinical impact of ReDS-guided management remains the next concern. Several prospective studies are underway. For example, we are currently comparing clinical outcomes between those who receive ReDS-guided congestion management by diuretic dose adjustment and those who receive diuretic adjustment without ReDS measurements. Another concern is the distribution of congestion. Pulmonary congestion is not necessarily associated with systemic congestion, which also has an independent prognostic impact [8]. Estimating plasma volume status by using several standard clinical parameters may also be useful in order to assess the degree of systemic congestion and estimate background etiologies [9]. Comorbidities including chronic kidney disease also cause congestive status in addition to heart failure. 

Another concern is how to intervene against pulmonary congestion. Traditionally, we adjust the dose of loop diuretics. However, a higher dose of loop diuretics is associated with worse clinical outcomes, due to intravascular hypovolemia, the stimulation of the renin–angiotensin system, and the worsening of renal function [2]. The use of a higher dose of loop diuretics causes diuretic resistance, which further deteriorates clinical outcomes. An accurate and repeated assessment of the lung fluid amount by utilizing ReDS systems in combination with other conventional modalities should be required to avoid the unnecessary up-titration of loop diuretics by repeated dose adjustments [4]. Another key should be to incorporate other medications that have diuretics effects, including vasopressin type 2 receptor antagonists, sodium–glucose cotransporter 2 receptors, and angiotensin receptor neprilysin inhibitors, to minimize the dose of loop diuretics to avoid drug-related adverse effects [1].

As discussed above, the ReDS system is not a perfect tool for assessing pulmonary congestion. The ReDS system is non-invasive and does not require expert techniques. On the contrary, it cannot distinguish pulmonary congestion from other similar conditions, including pneumonia, lung cancer, and acute respiratory distress syndrome. The ReDS system is generally useful for screening or follow-up of mild congestion. Further intensive tests are highly recommended in patients with high ReDS values. 
